# Basal forebrain cholinergic signaling in the basolateral amygdala promotes strength and durability of fear memories

**DOI:** 10.1038/s41386-022-01427-w

**Published:** 2022-09-02

**Authors:** Byron E. Crimmins, Nura W. Lingawi, Billy C. Chieng, Beatrice K. Leung, Stephen Maren, Vincent Laurent

**Affiliations:** 1 Decision Neuroscience Laboratory, School of Psychology, University of New South Wales, Sydney, NSW Australia; 2 Department of Psychological and Brain Sciences and Institute for Neuroscience, Texas A&M University, College Station, TX USA

**Keywords:** Fear conditioning, Extinction

## Abstract

The basolateral amygdala (BLA) complex receives dense cholinergic projections from the nucleus basalis of Meynert (NBM) and the horizontal limb of the diagonal band of Broca (HDB). The present experiments examined whether these projections regulate the formation, extinction, and renewal of fear memories. This was achieved by employing a Pavlovian fear conditioning protocol and optogenetics in transgenic rats. Silencing NBM projections during fear conditioning weakened the fear memory produced by that conditioning and abolished its renewal after extinction. By contrast, silencing HDB projections during fear conditioning had no effect. Silencing NBM or HDB projections during extinction enhanced the loss of fear produced by extinction, but only HDB silencing prevented renewal. Next, we found that systemic blockade of nicotinic acetylcholine receptors during fear conditioning mimicked the effects produced by silencing NBM projections during fear conditioning. However, this blockade had no effect when given during extinction. These findings indicate that basal forebrain cholinergic signaling in the BLA plays a critical role in fear regulation by promoting strength and durability of fear memories. We concluded that cholinergic compounds may improve treatments for post-traumatic stress disorder by durably stripping fear memories from their fear-eliciting capacity.

## Introduction

Post-traumatic stress disorder features obtrusive and incessant recalls of fear memories that were formed in response to one or more traumatic events [1, 2]. To improve clinical interventions, decades of work have focused on uncovering the mechanisms mediating the formation and inhibition of fear memories [3–5]. In the laboratory, this work has often been achieved by studying the extinction of Pavlovian fear conditioning [4, 6–8]. During fear conditioning, a fear memory is formed as an initially neutral stimulus is paired with a noxious event. During extinction, the fear memory comes to be inhibited as the stimulus is repeatedly presented on its own. A core finding is that the inhibition produced by extinction is unstable such that fear relapse after extinction occurs frequently [9]. One example of fear relapse includes the renewal effect [10–13], which restores fear responding by presenting the stimulus outside of its extinction context.

The basolateral amygdala (BLA) complex is a crucial site for the formation and extinction of fear memories [5, 8, 14–21]. Within the BLA, multiple lines of evidence emphasize the importance of the regulatory control exerted by local cholinergic inputs [22–28]. These inputs originate predominantly from two sub-territories of the basal forebrain, the nucleus basalis of Meynert (NBM) and the horizontal limb of the diagonal band of Broca (HDB) [29–31]. A recent study [24] showed that silencing the NBM to BLA cholinergic (^Ach^NBM→BLA) pathway during fear conditioning enhances the inhibition of fear produced by extinction. The opposite was found when this pathway was stimulated. Although this study convincingly demonstrates that BLA cholinergic signaling regulates fear memories, the breadth of this regulation and its underlying mechanisms remain unknown.

The present experiments sought to examine how basal forebrain cholinergic signaling in the BLA regulates the formation, extinction, and renewal of fear memories. We used optogenetics in transgenic rats to silence the ^Ach^NBM→BLA pathway or the HDB to BLA cholinergic (^Ach^HDB→BLA) pathway. Silencing of either pathway took place during fear conditioning or extinction. The long-term consequences of these manipulations were assessed during a post-extinction test and two retrieval tests in the extinction and conditioning contexts. Finally, we evaluated whether any behavioral change associated with silencing of the two cholinergic pathways was mediated by nicotinic acetylcholine receptors. This was achieved by systemic blockade of these receptors before fear conditioning or extinction.

## Materials and methods

See the Supplementary Material for detailed methods.

### Subjects

Experimentally naive female and male ChAT::Cre^+^ rats and Long-Evans rats were used. All rats were at least 8-week-old at the start of the experiments, and they were obtained from the breeding facility at the University of New South Wales (Sydney, Australia). The Animals Ethics Committee at the University of New South Wales approved all experimental procedures, which took place during the light cycle.

### Drugs

The nicotinic receptors antagonist mecamylamine (2.25 mg/kg) was injected intraperitoneally 20 min before the relevant experimental stage.

### Surgery

Rats received viral infusions in the NBM, HDB or BLA and optic fiber implants above the BLA. This was achieved under isoflurane anesthesia using standard stereotaxic procedures.

### Behavioral apparatus and procedures

Training and testing took place in Med Associates conditioning chambers. Two distinct physical contexts, A and B, were used. The auditory conditioned stimulus (CS) was a 30 s, 3 kHz pure tone (90 dB) and the unconditioned stimulus (US) was a 0.5 s, 0.8 mA foot shock. Fear conditioning involved 2, 3, or 4 CS-US pairings in context A. Fear extinction and test used 15 CS presentations in context B. The two within-subject retrieval tests included 10 or 5 CS presentations, with one test occurring in context A and the other in context B (order counterbalanced).

### Optogenetics

The inhibitory halorhodospsin (eNpHR3.0) was used to silence the cholinergic pathways and a null virus (eYFP) was used as control. Illumination was achieved through a 625 nm LED (Doric; at least 8 mW at the tip of the fiber optics) and started at the onset of the CS and ended 4 s after the CS terminated.

### Statistics

Freezing was the index of conditioned fear. It was rated in a time-sampling manner and judged as either freezing or not freezing every 2 s by a trained observer blind to the subjects’ group assignment. A proportion of the data was cross scored by a second naive observer; there was a high level of agreement between observers (Pearson product moment correlation >0.9). Planned orthogonal contrasts were used for the statistical analyses [32] and were conducted in the PSY software (School of Psychology, The University of New South Wales, Australia). For optogenetic experiments involving a four-group design (Figs. 2, 4, S2, S4), a first contrast tested for differences between the two eYFP groups (eYFP-ON and eYFP-OFF). A second contrast combined these two eYFP groups and tested for difference with the third control group eNpHR3.0-OFF. Finally, a third contrast combined the three control groups and tested for differences with the experimental eNpHR3.0-ON group. For optogenetic experiments involving a two-group design (Figs. 3, S3, 5D, S5B), a single contrast tested for differences between the two groups. For pharmacological experiments involving a four-group design (Figs. 5B, S5A), the contrasts used were similar to a two-way ANOVA and used two main factors (training strength and treatment) and their interactions. Retrieval tests included the within-subject factor of context identity (i.e., context A vs. context B). Within-session changes in freezing were assessed by planned linear trend analyses. The Type I error rate was controlled at alpha = 0.05 for each contrast tested. If interactions were detected, follow-up simple effects analyses were calculated to determine the source of the interactions. There were no sex differences in any of the analyses. For simplicity, the main Figures only report freezing data during the first five trials of the post-extinction test and retrieval tests. Complete data are available in the Supplementary Material.

**Fig. 1:**
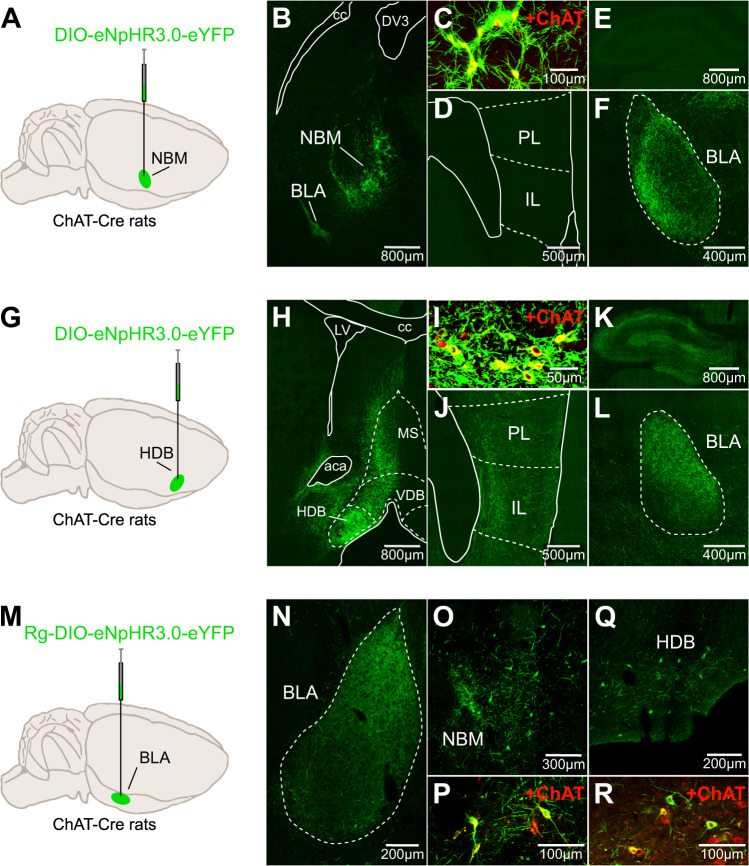
NBM and HDB cholinergic neurons project to the BLA. **A** ChAT::Cre^+^ rats were unilaterally infused in the NBM with DIO-eNpHR3.0-eYFP. **B** Micrograph showing NBM viral expression. **C** Viral expression was restricted to NBM cholinergic neurons. ChAT choline acetyltransferase. **D**–**F** NBM cholinergic neurons project to the BLA but not to the prelimbic cortex (PL), the infralimbic cortex (IL) or dorsal hippocampus. **G** ChAT::Cre^+^ rats were unilaterally infused in the HDB with DIO-eNpHR3.0-eYFP. **H** Micrograph showing viral expression in HDB and minimal expression in the medial septum (MS) or vertical limb of the diagonal band (VDB). **I** Viral expression was restricted to HDB cholinergic neurons. **J**–**L** HDB cholinergic neurons project to PL, IL, dorsal hippocampus, and BLA. **M** ChAT::Cre^+^ rats were unilaterally infused in the BLA with Rg-DIO-eNpHR3.0-eYFP. **N** Micrograph showing BLA viral expression. **O**–**R** Viral expression was detected in NBM and HDB neurons, which were cholinergic.

**Fig. 2:**
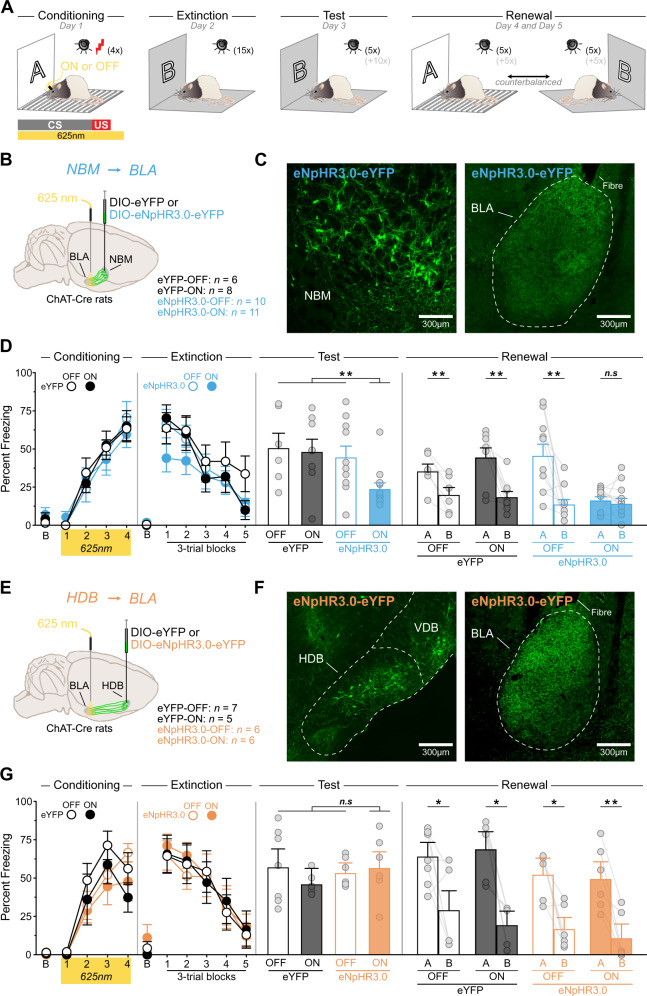
^Ach^NBM→BLA silencing during fear conditioning enhances extinction and prevents renewal. **A** Schematic representation of the experimental design. **B** ChAT::Cre^+^ rats were bilaterally infused in the NBM with either DIO-eYFP or DIO-eNpHR3.0-eYFP and fiber optics were bilaterally implanted above the BLA. **C** Micrographs showing DIO-eNpHR3.0-eYFP expression in NBM cholinergic neurons (left), eYFP-positive BLA cholinergic terminals and fiber optic placements (right). **D** Fear conditioning was similar in all groups. Baseline freezing (B) during conditioning was similar in all groups (smallest *p* = 0.19). Extinction was similar in all groups. Baseline freezing (B) during extinction was similar in all groups (smallest *p* = 0.21). ^Ach^NBM→BLA silencing reduced freezing during the post-extinction test. Baseline freezing at test was similar in all groups (smallest p = 0.33). ^Ach^NBM→BLA silencing abolished fear renewal. Baseline freezing during the retrieval tests is described in Fig. S2. **E** ChAT::Cre^+^ were bilaterally infused in the HDB with either DIO-eYFP or DIO-eNpHR3.0-eYFP and fiber optics were bilaterally implanted above the BLA. **F** Micrographs showing DIO-eNPHR3.0-eYFP expression in HDB cholinergic neurons (left), eYFP-positive BLA cholinergic terminals and fiber optic placements (right). **G** Fear conditioning was similar in all groups. Baseline freezing (B) during conditioning was similar in all groups (smallest *p* = 0.49). Extinction was similar in all groups. Baseline freezing (B) during extinction was similar in all groups (smallest *p* = 0.06). All rats displayed similar freezing during the post-extinction test. Baseline freezing at test was similar in all groups (smallest *p* = 0.11). ^Ach^HDB→BLA silencing had no effect on fear renewal. Baseline freezing during the retrieval tests is described in Fig. S2. Data are shown as mean ± SEM. Asterisks denote significant effect (*p < 0.05; **p < 0.01; ***p < 0.001). n.s. nonsignificant. Each light gray dot corresponds to one animal.

**Fig. 3:**
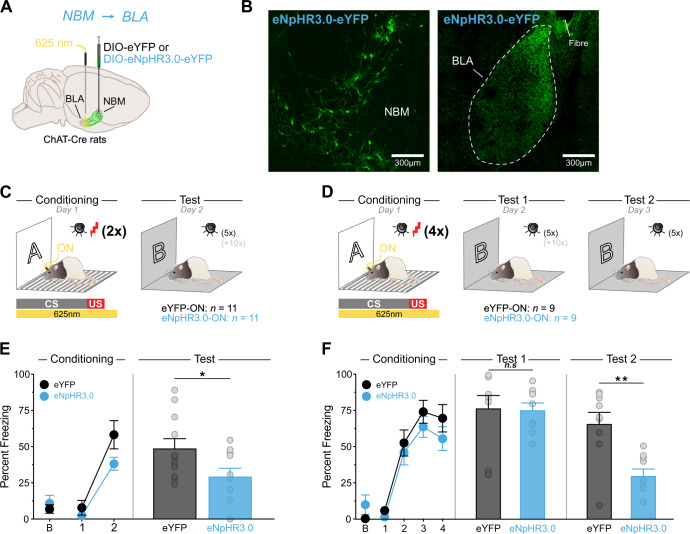
^Ach^NBM→BLA silencing during fear conditioning weakens the acquisition of a fear memory. **A** ChAT::Cre^+^ rats were bilaterally infused in the NBM with either DIO-eYFP or DIO-eNpHR3.0-eYFP and fiber optics were bilaterally implanted above the BLA. **B** Micrographs showing DIO-eNpHR3.0-eYFP expression in NBM cholinergic neurons (left), eYFP-positive BLA cholinergic terminals and fiber optic placements (right). **C** Schematic representation of the experimental design for weak fear conditioning. **D** Schematic representation of the experimental design used for strong fear conditioning. **E** Weak fear conditioning was similar in all groups. Baseline freezing (B) during conditioning was similar in both groups (*p* = 0.45). ^Ach^NBM→BLA silencing reduced freezing at test. Baseline freezing at test was similar in both groups (*p* = 0.81). **F** Strong fear conditioning was similar in all groups (*p* = 0.42). Baseline freezing (B) during conditioning was similar in both groups (*p* = 0.17). During test 1, rats displayed similar freezing. Baseline freezing during test 1 was similar in both groups (*p* = 0.84). ^Ach^NBM→BLA silencing during strong fear conditioning reduced freezing at test 2. Baseline freezing during test 2 was similar in both groups (*p* = 0.24). Data are shown as mean ± SEM. Asterisks denote significant effect (*p < 0.05; **p < 0.01; ***p < 0.001). n.s. nonsignificant. Each light gray dot corresponds to one animal.

## Results

### NBM and HDB cholinergic neurons project to the BLA

We first examined the connectivity of NBM and HDB cholinergic neurons in transgenic rats expressing Cre recombinase in choline acetyltransferase neurons (ChAT::Cre^+^; [33]). Rats were infused in the NBM (Fig. 1A, B) or the HDB (Fig. 1G, H) with a Cre dependent adeno-associated virus encoding the neuronal silencer Halorhodopsin and the fluorophore eYFP (eNpHR3.0 virus). Viral expression was restricted to cholinergic neurons (Figs. 1C, I, S1A–D). The NBM infusion resulted in eYFP-positive terminals in the BLA (Fig. 1F) but not in the prelimbic cortex, the infralimbic cortex or dorsal hippocampus (Fig. 1D, E). By contrast, the HDB infusion produced eYFP-positive terminals in all four brain regions (Fig. 1J–L). Next, we employed a retrograde approach by infusing in the BLA (Fig. 1M, N) the eNpHR3.0 virus in a retrograde serotype. We found eYFP-positive neurons in the NBM (Fig. 1O) and the HDB (Fig. 1Q), which were cholinergic (Fig. 1P, R). We also found that BLA cholinergic innervation originated from the HDB and not the adjacent and cholinergic-rich medial septum (MS) or vertical limb of the diagonal band (Fig. S1E). Accordingly, MS infusion of the eNpHR3.0 virus did not yield BLA eYFP-positive terminals (Fig. S1F–M). These results agree with the literature [29–31] and indicate that although NBM and HDB cholinergic neurons differ in their anatomical targets, both innervate the BLA.

**Fig. 4:**
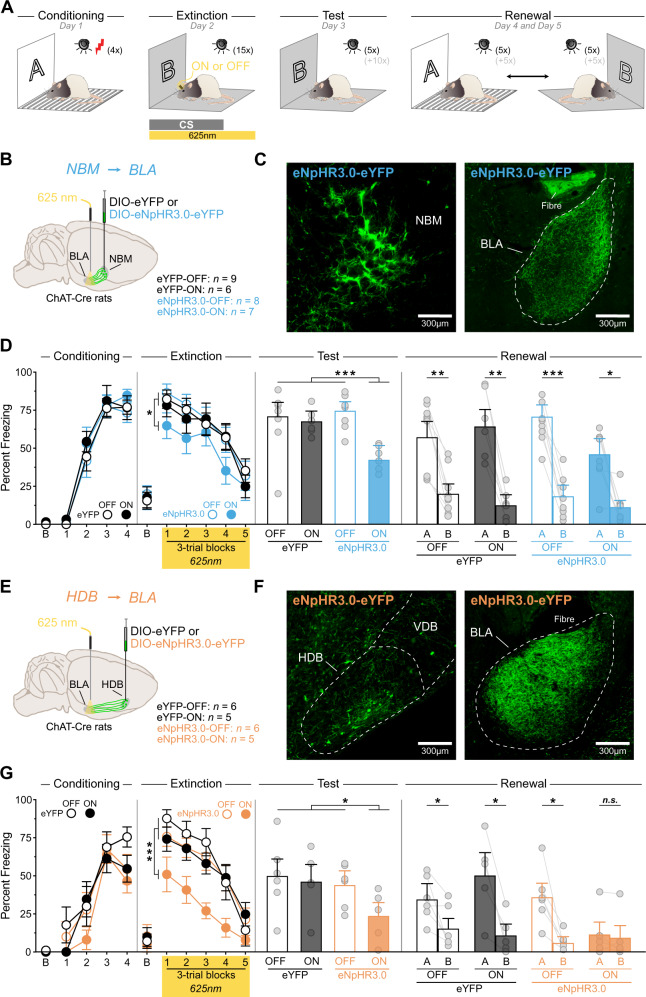
^Ach^HDB→BLA silencing during extinction enhances extinction and prevents renewal. **A** Schematic representation of the experimental design. **B** ChAT::Cre^+^ rats were bilaterally infused in the NBM with either DIO-eYFP or DIO-eNpHR3.0-eYFP and fiber optics were bilaterally implanted above the BLA. **C** Micrographs showing DIO-eNpHR3.0-eYFP expression in NBM cholinergic neurons (left), eYFP-positive BLA cholinergic terminals and fiber optic placements (right). **D** Fear conditioning was similar in all groups (smallest *p* = 0.30). Baseline freezing (B) during conditioning was similar in all groups (smallest *p* = 0.11). ^Ach^NBM→BLA silencing reduced freezing during extinction. Baseline freezing (B) during extinction was similar in all groups (smallest *p* = 0.65). ^Ach^NBM→BLA silencing reduced freezing during the post-extinction test. Baseline freezing at test was similar in all groups (smallest p = 0.33). ^Ach^NBM→BLA silencing had no effect on renewal. Baseline freezing during the retrieval tests is described in Fig. S4. **E** ChAT::Cre^+^ rats were bilaterally infused in the HDB with either DIO-eYFP or DIO-eNpHR3.0-eYFP and fiber optics were bilaterally implanted above the BLA. **F** Micrographs showing DIO-eNpHR3.0-eYFP expression in HDB cholinergic neurons (left), eYFP-positive BLA cholinergic terminals and fiber optic placements (right). **G** Fear conditioning was similar in all groups. Baseline freezing (B) during conditioning was similar in all groups (smallest *p* = 0.27). ^Ach^HDB→BLA silencing reduced freezing during. Baseline freezing (B) during extinction was similar in all groups (smallest *p* = 0.60). ^Ach^HDB→BLA silencing reduced freezing during the post-extinction test. Baseline freezing at test was similar in all groups (smallest *p* = 0.21). ^Ach^NBM→BLA silencing abolished fear renewal. Baseline freezing during the retrieval tests is described in Fig. S4. Data are shown as mean ± SEM. Asterisks denote significant effect (*p < 0.05; **p < 0.01; ***p < 0.001). n.s. nonsignificant. Each light gray dot corresponds to one animal.

### ^Ach^NBM→BLA silencing during fear conditioning weakens the acquisition of a fear memory and prevents renewal

Having established that NBM and HDB cholinergic neurons project to the BLA, we sought to determine if these projections regulate fear memories. The behavioral protocol (Fig. 2A) started with fear conditioning during which an auditory conditioned stimulus (CS) was paired with a foot shock unconditioned stimulus (US) in context A. Fear to the CS was then extinguished in a distinct context B and was later tested in that context. Next, two retrieval tests were given: one in which the CS was presented in context A and the other where it was presented in context B. These tests allowed assessing the capacity of the fear memory to renew after extinction. Indeed, an extinguished fear memory typically fails to elicit fear in its extinction context (e.g., context B), but it does so outside of that context (e.g., context A) [10–13].

We first assessed the impact of ^Ach^NBM→BLA silencing during fear conditioning. ChAT::Cre^+^ rats were infused in the NBM with the eNpHR3.0 virus or a control virus encoding eYFP only (eYFP virus), and they were implanted with fiber optics above the BLA (Figs. 2B, C, S2A–C). A subset of rats in each viral condition underwent the CS-US pairings under light illumination (ON). Light illumination was omitted in the remaining rats (OFF). Thus, ^Ach^NBM→BLA was silenced in eNpHR3.0-ON rats and was left intact in the other control rats (eYFP-OFF, eYFP-ON, eNpHR3.0-OFF). The freezing data are presented in Figs. 2D, S2D. Fear conditioning was successful and freezing gradually increased across trials (*F*_1,31_ = 125.1, *p* < 0.001), regardless of groups (smallest *p* = 0.65). Extinction was also successful and freezing gradually decreased across trials (*F*_1,31_ = 94.80, *p* < 0.001), regardless of groups (smallest *p* = 0.05). ^Ach^NBM→BLA silencing reduced freezing during the post-extinction test relative to the control rats (*F*_1,31_ = 9.76, *p* < 0.01), which displayed equivalent freezing (smallest *p* = 0.58). The silencing also abolished fear renewal (*F*_1,31_ = 10.44, *p* < 0.01). The control rats displayed similar freezing (smallest *p* = 0.54) and froze more in context A than in context B (eYFP-OFF: *F*_1,5_ = 16.99, *p* < 0.01; eYFP-ON: *F*_1,7_ = 15.41, *p* < 0.01; eNpHR3.0-OFF: *F*_1,9_ = 14.69, *p* < 0.01). By contrast, rats with ^Ach^NBM→BLA silencing froze very little in both contexts (*p* = 0.60). Separate analyses (Fig. S2D) revealed that all rats froze more in context A than in context B at the beginning (i.e., pre-CS period) of the retrieval tests, indicating that ^Ach^NBM→BLA silencing did not prevent the rats from discriminating the two contexts. Importantly, renewal in context A was associated with a substantial increase in freezing to the CS relative to pre-CS freezing levels (Fig. S2D).

Next, we examined the impact of ^Ach^HDB→BLA silencing during fear conditioning (Figs. 2E, F, S2E–G). The freezing data are presented in Figs. 2G, S2H. Fear conditioning was successful and freezing gradually increased across trials (*F*_1,20_ = 89.05, *p* < 0.001), regardless of groups (smallest *p* = 0.20). Extinction was also successful and freezing gradually decreased across trials (*F*_1,20_ = 92.25, *p* < 0.001), regardless of groups (smallest *p* = 0.69). ^Ach^HDB→BLA silencing during fear conditioning had no effect during the post-extinction test and all rats displayed similar freezing (smallest *p* = 0.30). The silencing had also no effect on renewal (*p* = 0.08). All rats froze more in context A than in context B (eYFP-OFF: *F*_1,6_ = 6.98, *p* < 0.05; eYFP-ON: *F*_1,4_ = 12.82, *p* < 0.05; eNpHR3.0-OFF: *F*_1,5_ = 14.69, *p* < 0.05; eNpHR3.0-ON: *F*_1,5_ = 23.78, *p* < 0.001). Together, these data show that ^Ach^NBM→BLA silencing during fear conditioning enhanced the loss of fear produced by extinction, as fear was reduced in the post-extinction test. They also show that this silencing prevented the renewal of fear typically observed after extinction. By contrast, ^Ach^HDB→BLA silencing during fear conditioning had no effect across extinction, the post-extinction test, and the retrieval tests.

Because ^Ach^NBM→BLA silencing occurred during fear conditioning, we reasoned that the accelerated loss of fear produced by extinction revealed that the fear memory entered extinction in a weaker state. To test this possibility, we employed a similar approach as before (Figs. 3A, B, S3A–C), except that rats received ^Ach^NBM→BLA silencing while undergoing a weaker fear conditioning protocol (2 × CS-US; Fig. 3C). In this protocol (Figs. 3E, S3D), ^Ach^NBM→BLA silencing had no immediate effect, and freezing gradually increased across the fear conditioning trials (*F*_1,20_ = 77.26, *p* < 0.001), regardless of groups (*p* = 0.15). However, the silencing reduced freezing at test the following day (*F*_1,20_ = 4.71, *p* < 0.05). Thus, a weak fear memory was more sensitive to ^Ach^NBM→BLA silencing, resulting in a deficit at an earlier time point. To further confirm this finding, we replicated our previous results using the stronger fear conditioning protocol (4 × CS-US; Figs. 3D, S3E, F). In this protocol (Figs. 3F, S3G), ^Ach^NBM→BLA silencing had no immediate effect, and freezing gradually increased across the fear conditioning trials (*F*_1,16_ = 101.13, *p* < 0.001), regardless of groups (*p* = 0.42). The silencing did not result in fear reduction during a test conducted the day after fear conditioning (*p* = 0.90). However, this reduction was simply delayed, as it was detected in a subsequent test (*F*_1,16_ = 14.34, *p* < 0.01). These results therefore indicate that ^Ach^NBM→BLA silencing during fear conditioning weakens the acquisition of a fear memory and that the capacity to observe this weakening depends on the strength of the original memory.

### ^Ach^HDB→BLA silencing during extinction enhances extinction and renewal

The roles played by the ^Ach^NBM→BLA and ^Ach^HDB→BLA pathways during fear extinction is unknown. To address this gap, we employed the same approach as before (Figs. 4A–C, 4E, F, S4A–C, E–G), except that silencing occurred during the CS alone presentations in extinction. The freezing data for ^Ach^NBM→BLA silencing during extinction are presented in Figs. 4D, S4D. Fear conditioning was successful and freezing gradually increased across trials (*F*_1,26_ = 360.77, *p* < 0.001), regardless of groups (smallest *p* = 0.30). Extinction was also successful and freezing gradually decreased across trials (*F*_1,26_ = 172.94, *p* < 0.001). However, ^Ach^NBM→BLA silencing reduced freezing during extinction relative to control rats (*F*_1,26_ = 5.67, *p* < 0.05), which displayed equivalent freezing (smallest p = 0.74). Yet, the decrease in freezing was similar across groups (smallest *p* = 0.28). ^Ach^NBM→BLA silencing also reduced freezing during the post-extinction test relative to the control rats (*F*_1,26_ = 21.85, *p* < 0.001), which displayed equivalent freezing (smallest *p* = 0.40). Nevertheless, the silencing had no effect on renewal (*p* = 0.06), and all rats froze more in context A than in context B (eYFP-OFF: *F*_1,8_ = 31.48, *p* < 0.001; eYFP-ON: *F*_1,5_ = 48.08, *p* < 0.001; eNpHR3.0-OFF: *F*_1,7_ = 74.77, *p* < 0.001; eNpHR3.0-ON: *F*_1,6_ = 13.56, *p* < 0.01).

The freezing data for ^Ach^HDB→BLA silencing during extinction are presented in Figs. 4G, S4D. Fear conditioning was successful and freezing gradually increased across trials (*F*_1,18_ = 68.80, *p* < 0.001), regardless of groups (smallest *p* = 0.62). Extinction was also successful and freezing gradually decreased across trials (*F*_1,18_ = 158.81, *p* < 0.001). However, ^Ach^HDB→BLA silencing reduced freezing during extinction relative to control rats (*F*_1,18_ = 21.79, *p* < 0.001), which displayed equivalent freezing (smallest p = 0.52). Yet, the decrease in freezing was similar across groups (smallest *p* = 0.06). ^Ach^HDB→BLA silencing also reduced freezing during the post-extinction test relative to the control rats (*F*_1,18_ = 5.31, *p* < 0.05), which displayed equivalent freezing (smallest *p* = 0.69). Importantly, the silencing also abolished renewal (*F*_1,18_ = 10.91, *p* < 0.01). The control rats displayed equivalent freezing (smallest *p* = 0.06) and froze more in context A than in context B (eYFP-OFF: *F*_1,5_ = 8.41, *p* < 0.01; eYFP-ON: *F*_1,4_ = 19.11, *p* < 0.05; eNpHR3.0-OFF: *F*_1,5_ = 15.72, *p* < 0.05). By contrast, rats with ^Ach^HDB→BLA silencing froze very little in both contexts (*p* = 0.46). Separate analyses revealed that all rats froze more in context A than in context B at the beginning (i.e., pre-CS period) of the retrieval tests (Fig. S4H), indicating that ^Ach^HDB→BLA silencing did not impair the capacity of the rats to discriminate the two contexts. Together, these results indicate that ^Ach^NBM→BLA and ^Ach^HDB→BLA silencing during extinction enhanced the loss of fear produced by extinction, as fear was reduced in the post-extinction test. They also dissociate the long-term effects of the two silencing, as only ^Ach^HDB→BLA silencing was found to abolish renewal of the extinguished fear memory.

### Systemic nicotinic receptors blockade mimics the effects of ^Ach^NBM→BLA silencing

**Fig. 5:**
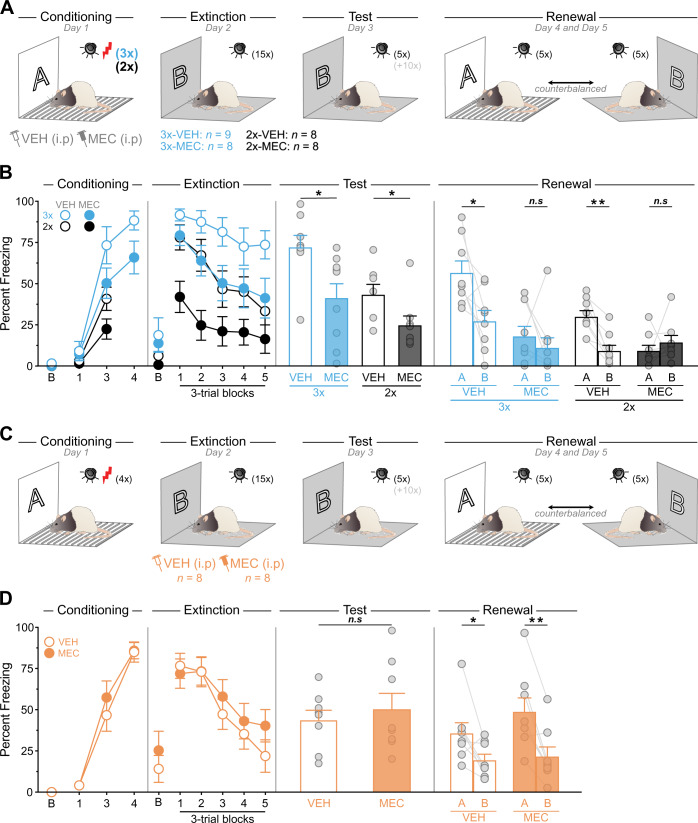
Systemic nicotinic receptors blockade mimics the effects of ^Ach^NBM→BLA silencing. **A** Schematic representation of the experimental design. **B** Fear conditioning was successful in all groups. Baseline freezing (B) during conditioning was similar in all groups (smallest *p* = 0.20). During extinction, strong fear conditioning produced higher freezing than weak fear conditioning and MEC reduced freezing. Baseline freezing (B) during extinction was higher in the strongly-trained rats (*F*_1,29_ = 4.64; *p* < 0.05) but was not influenced by MEC (*p* = 0.40). During the post-extinction test, strongly-trained rats froze more than weakly-trained rats and MEC-treated rats froze less than VEH-treated rats. Baseline freezing (B) at test was similar in all groups (*p* = 0.32). During the retrieval tests, strongly-trained rats and VEH-treated rats froze more than weakly-trained rats and MEC-treated rats, respectively. Critically, fear renewal was abolished by MEC. Baseline freezing during the retrieval tests is described in Fig. S5. **C** Schematic representation of the experimental design. **D** Fear conditioning was similar in both groups. Baseline freezing (B) during conditioning was similar in both groups (*p* = 0.55). Extinction was similar in both groups. Baseline freezing (B) during extinction was similar in both groups (*p* = 0.44). During the post-extinction test, rats displayed similar freezing. Baseline freezing at test was similar in both groups (smallest *p* = 0.14). Fear renewal was observed in all rats regardless of treatment. Baseline freezing during the retrieval tests is described in Fig. S5. Data are shown as mean ± SEM. Asterisks denote significant effect (*p < 0.05; **p < 0.01; ***p < 0.001). n.s. nonsignificant. Each light gray dot corresponds to one animal.

Evidence suggests that the ^Ach^NBM→BLA pathway regulates fear memories via activation of nicotinic acetylcholine receptors (nAchR) [24]. We therefore examined the effects of nAchR blockade in fear regulation. Rats received a strong (3 CS × US; 3×) or weak (2 CS × US; 2×) fear conditioning protocol under systemic administration of vehicle (VEH) or the nAchR antagonist mecamylamine (MEC; Fig. 5A). The freezing data are presented in Figs. 5B, S5A. Fear conditioning was successful and freezing gradually increased across trials in the weak (*F*_1,14_ = 39.43, *p* < 0.001) and strong (*F*_1,15_ = 98.03, *p* < 0.001) fear conditioning protocol, irrespective of treatment (smaller *p* = 0.09). Extinction was also successful and freezing gradually decreased across trials (*F*_1,29_ = 49.64, *p* < 0.001). As expected, the strong protocol produced higher freezing than the weak protocol during extinction (*F*_1,29_ = 31.63, *p* < 0.001). Further, MEC prior to conditioning reduced freezing across extinction (*F*_1,29_ = 18.32, *p* < 0.001). Although the gradual decline in freezing was similar regardless of the fear conditioning protocol or pharmacological treatment (smallest *p* = 0.58), it was stronger in MEC-treated rats submitted to the weak protocol (*F*_1,29_ = 4.97, *p* < 0.05). A similar pattern of results was obtained during the post-extinction test. Strongly-trained rats froze more than weakly-trained rats (*F*_1,29_ = 9.58; *p* < 0.01). MEC-treated rats froze less than VEH-treated rats (*F*_1,29_ = 11.41; *p* < 0.01), regardless of the fear conditioning protocol (*p* = 0.41). During the retrieval tests, strongly-trained rats and VEH-treated rats froze more than weakly-trained rats (*F*_1,29_ = 11.35; *p* < 0.01) and MEC-treated rats (*F*_1,29_ = 22.13; *p* < 0.001), respectively. Critically, fear renewal was abolished by MEC (*F*_1,29_ = 9.22; *p* < 0.01) regardless of the fear conditioning protocol (*p* = 0.20). Thus, renewal was present in VEH-treated rats (weak: F_1,7_ = 21.36; p < 0.01; strong: *F*_1,7_ = 7.04; *p* < 0.05) but not MEC-treated rats (weak: *p* = 0.44; strong: *p* = 0.43). Next, we examined the effects of nAchR antagonism prior to extinction (Fig. 5C). Freezing data are presented in Fig. 5D. Fear conditioning was successful and freezing gradually increased across trials (*F*_1,14_ = 486.61; *p* < 0.001), irrespective of group (*p* = 0.91). Extinction was also successful and freezing gradually decreased across trials (*F*_1,14_ = 793.44; *p* < 0.001), irrespective of group (*p* = 0.08). During the post-extinction test, rats displayed substantial and similar freezing regardless of treatment (*p* = 0.57). Finally, fear renewal was observed in all rats regardless of treatment (*p* = 0.35; VEH: *F*_1,7_ = 6.22; *p* < 0.05; MEC: *F*_1,7_ = 22.84, *p* < 0.01). Taken together, these results indicate that the effects of ^Ach^NBM→BLA silencing during fear conditioning can be reproduced by nAchR blockade before fear conditioning. By contrast, the effects of ^Ach^HDB→BLA silencing during extinction cannot be reproduced by nAchR blockade before fear extinction.

## Discussion

Considerable research effort has been deployed to describe the psychological and neural mechanisms underlying fear regulation [3–5]. The present study used optogenetics and pharmacological methods to examine how basal forebrain cholinergic projections to the BLA influence the formation, extinction, and renewal of fear memories. We revealed for the first time that these projections control the strength and durability of fear memories. This control was implemented by NBM cholinergic projections during fear memory formation and was supported by HDB cholinergic projections during extinction. Removing either form of cholinergic control produced a fear memory that lost its fear-eliciting capacity faster following extinction and that was unable to trigger fear again despite context shifts. We also gathered evidence suggesting that NBM-mediated control involved nAchR activation during fear memory formation whereas HDB-mediated control during extinction did not recruit these receptors. Together, these findings underscore the critical role played BLA cholinergic signaling in fear regulation.

Previous research showed that ^Ach^NBM→BLA silencing during fear conditioning accelerates the loss of fear produced by extinction [24]. We confirmed this finding and provided novel insights into its long-term consequences and underlying mechanisms. We found that ^Ach^NBM→BLA silencing during fear conditioning abolished fear renewal, implying that this silencing durably hinders the fear-eliciting capacity of a fear memory. We also demonstrated that extinction is not required to observe fear reduction following ^Ach^NBM→BLA silencing. This reduction was uncovered by diminishing the number of CS-US pairings across fear conditioning, suggesting that the primary function of NBM cholinergic projections to the BLA is to promote fear memory strength. This function likely involves activation of BLA nAchR, as systemic blockade of these receptors before fear conditioning mimicked the results obtained with ^Ach^NBM→BLA silencing. It will be essential for future studies to replicate this finding using nAchR blockade restricted to the BLA. Interestingly, ^Ach^HDB→BLA silencing during fear conditioning left the formation, extinction, and renewal of a fear memory intact. This indicates that the two cholinergic pathways are not functionally redundant.

^Ach^NBM→BLA silencing during extinction produced an immediate reduction in fear that persisted across a later test. The same outcome was obtained with ^Ach^HDB→BLA silencing during extinction. The source of these effects remains to be identified and could be driven by weakening the fear memory or an enhancement of extinction. Future studies examining the role of this pathway during memory reconsolidation [34–37] may help clarify which interpretation is correct. Regardless, we also found that fear renewal was preserved by ^Ach^NBM→BLA silencing during extinction but was abolished by ^Ach^HDB→BLA silencing during extinction. These results further confirm that the two cholinergic pathways are not functionally redundant. They also indicate that NBM cholinergic control of BLA activity during extinction does not regulate the durability of fear memories, contrasting with its capacity to achieve such regulation during fear conditioning. However, they do suggest that this regulation can be implemented by HDB cholinergic projections to the BLA during extinction. Importantly, this regulation does not appear to recruit activation of BLA nAchR, as systemic blockade of these receptors before extinction left intact the formation, extinction, and renewal of a fear memory. Accordingly, we speculate that HDB-mediated control of BLA activity may be mediated by local muscarinic acetylcholine receptors. Consistent with this, systemic administration of the muscarinic antagonist, scopolamine, has been shown to reduce freezing during fear extinction and impair later retrieval [38]. Future studies should aim to replicate these results using scopolamine infusion in the BLA. Together, our findings point to the critical role played by ^Ach^HDB→BLA pathway during fear extinction, a role consistent with HDB connectivity to the BLA, the infralimbic cortex and the hippocampus, which are part of neuronal circuitry supporting extinction [5, 12, 18–20, 39–43].

Three aspects of our results are worth considering. The first is that none of the results reported here can be explained in terms of abnormal behavior in ChAT::Cre^+^ rats. Although such abnormalities have been reported [44], they do not include the freezing response. Further, ChAT::Cre^+^ rats were used in both the control and experimental groups in our experiments. The second aspect to consider is that silencing cholinergic pathways did not prevent discrimination between the experimental contexts. All rats displayed more fear in the conditioning context than in the extinction context at the beginning of the retrieval tests (Supplementary Material). Thus, basal forebrain cholinergic signaling in the BLA does not appear to be involved in context processing. It is noteworthy that discrimination between the two contexts was absent in our pharmacological experiments, but this was true regardless of treatments. The third and final aspect to consider is that the lack of renewal was not due to our manipulations reducing fear prior to the renewal test. In all experiments, rats exited the post-extinction test with equivalent fear (Supplementary Material). Further, ^Ach^NBM->BLA silencing during fear extinction reduced fear during the post-extinction test but spared subsequent fear renewal. Finally, the weakly-trained rats treated with vehicle displayed levels of test fear that were equivalent to those shown by strongly-trained rats treated with mecamylamine. Yet, the former rats exhibited fear renewal whereas the latter did not. We are therefore confident with our interpretation that the lack of renewal in our experiments indicates that disrupting BLA cholinergic signaling hinders the durability of fear memories.

Renewal and other fear restoration phenomena are taken as evidence that extinction is not forgetting but rather, involves the formation of an inhibitory memory that competes with the fear memory for behavioral control [6, 7, 9, 45]. Yet, fear restoration phenomena also suggest that some protective mechanisms are in place to ensure that a fear memory is inhibited and not forgotten across extinction. We propose that these mechanisms involve cholinergic regulation of BLA activity by the basal forebrain and that in their absence, a fear memory is forgotten instead of being inhibited across extinction. That is, we take the lack of renewal in our experiments as evidence of forgetting. This proposal is more suitable to the results obtained following ^Ach^NBM→BLA silencing during fear conditioning, as we demonstrated that this silencing directly interferes with fear memory formation. By contrast, we cannot exclude the possibility that the absence of renewal produced by ^Ach^HDB→BLA silencing during fear extinction reflected an enhancement of extinction. Regardless, the validity of our proposal could be tested based on its various underlying predictions. For example, it predicts that disrupting basal forebrain cholinergic signaling in the BLA could eliminate all fear restoration phenomena and potentially reverse the enduring physiological changes encoding fear memories in the BLA.

In conclusion, basal forebrain cholinergic projections to the BLA play a critical role in fear regulation by promoting the strength and durability of fear memories. These functions were supported during fear conditioning and extinction by projections arising from the NBM and the HDB, respectively. NBM-mediated regulation recruited nAchR activation whereas HDB-mediated regulation did not. Overall, our findings suggest that cholinergic compounds could be used to durably strip fear memories from their fear-eliciting capacity, which have clinical implications for the treatment of post-traumatic stress disorder.

## Supplementary information

Supplemental Material

Freezing Data
