# Supplementary material for: Basal forebrain cholinergic signaling in the basolateral amygdala promotes strength and durability of fear memories
Source: Neuropsychopharmacology. 2022 Sep 2;48(4):605–14. doi: 10.1038/s41386-022-01427-w (PMC9938249; doi:10.1038/s41386-022-01427-w)
Supplement: Supplementary file 1 — Supplemental Material [file 41386_2022_1427_MOESM1_ESM.pdf]

Supplementary Methods and Materials

Subjects

The subjects were experimentally naive female and male ChAT::Cre^+ rats (*n* = 188; Rat Resource & Research Centre, USA; #00658; [1]) bred in a Long-Evans background or Long-Evans rats (*n* = 49). The rats were obtained from the Breeding Facility at the University of New South Wales (Sydney, Australia) and were 8-12 weeks old at the start of the experiments. They were housed in plastic boxes (3-4 rats per box) located in a climate-controlled colony room maintained on a 12 h light/dark cycles (lights on between 7:00 A.M. and 7.00 P.M.). The rats were handled daily four days prior to the start of the behavioral procedures. Histological assessments prevented the inclusion of an equal number of females and males in each experimental group. However, separate statistical analyses failed to reveal sex differences during the various experimental stages (smallest p=0.17). The Animals Ethics Committee at the University of New South Wales approved all experimental procedures, which took place during the light cycle.

Drugs

Mecamylamine hydrochloride (Sigma-Aldrich Australia; # M9020), a non-selective and non-competitive antagonist of nicotinic acetylcholine receptors was diluted in 0.9% saline to reach a final concentration of 2.25 mg/mL. Rats received systemic intraperitoneal (i.p.) injections at 1 mL/kg 20 min before the relevant experimental stage. 0.9% saline was used as the control solution.

Viruses

The following adeno-associated viruses (AAV) were used: AAV5-EF1a-DIO-eNpHR3.0-eYFP (eNpHR3.0; Addgene; #26966), AAV5-Ef1a-DIO-EYFP (Addgene; #27056; eYFP) and RgAAV2-hSyn-eNpHR3.0-eYFP (Rg-DIO-eNpHR3.0; Vector and Genome Engineering Facility located, Westmead, Australia).

Surgery

At the time of surgery, male rats weighted between 250 and 420 g whereas female rats weighted between 200 and 320 g. A continuous flow of mixed isoflurane and oxygen gas was used to anesthetize rats, which were then placed in a stereotaxic frame (Kopf Instruments; California, USA). An incision was made to expose the scalp and the incisor bar was adjusted to align bregma and lambda on the same horizontal plane. For viral infusions, holes were drilled bilaterally into the skull above the targeted brain region at the following coordinates (indicated in mm relative to bregma; male/female when necessary): NBM: - 1.4 antero-posterior (AP), ± 3.2 medial-lateral (ML), - 7.25/7.15 dorsal-ventral (DV); HDB: + 0.9 AP, ± 0.65 ML, - 8.45/8.37 DV; BLA: -2.45 AP, ± 5 ML, - 9.25/9.15 DV. Infusions were conducted using a 1 µL Hamilton syringe attached to an infusion pump (Pump 11 Elite Nanometer, Harvard Apparatus). A total of 0.5 µL of the adequate AAV was infused in each hemisphere at a rate of 0.1 µL/min. The syringe was left in place for additional 10 min to allow for diffusion. At the end of the infusion, the incision was closed by using wound-closure clips (EZ Clip, Stoelting, USA). All rats were given a 0.4 mL i.p. injection of procaine penicillin solution (150 mg/kg; Duplocillin) after the surgery. Animals were allowed to recover for 7 d before the behavioral procedures.

When required, a second surgery was performed three weeks later to implant fiber optics (Doric Lenses, Quebec, Canada; MFC_400/430-0.48_10mm_RM2_FLT). The procedure was like that described before. The coordinates used for the BLA were the following: -2.45 AP, ± 5 ML, - -8.4/-8.3 DV. The fiber optics were maintained in position with dental cement. Rats were allowed to recover for 7 d before the behavioral procedures.

Apparatus

Training and testing took place in Med Associates conditioning chambers (Vermont, USA) enclosed in sound- and light-resistant shells. The floor of the chambers consisted of stainless-steel rods (3.8 mm in diameter, spaced 16 mm apart) that could be connected to a constant current generator to deliver a 0.5 s x 0.8 mA intensity foot shock unconditioned stimulus (US). A 3 W, 28 V house light provided illumination of the operant chamber. Each chamber contained a speaker connected to a sound card to generate a 3 kHz, 90 dB pure tone conditioned stimulus (CS). The CS lasted 30 s in all experiments. Each chamber was equipped with infra-red cameras to record the rat's behavior. A computer located in another room in the laboratory controlled the delivery of stimuli via Med-PC V Software Suite. Two distinct physical contexts (A and B) were used. Context A involved the standard and bared operant chambers with the house light off at all time and stainless grid floor. Context B had the house light off, a smooth black Perspex floor, striped walls, and peppermint odor.

Behavioral procedures

On day 1, rats received two pre-exposure sessions that occurred at least two hours apart. One session took place in context A and the other in context B (counterbalanced). Each session lasted approximately 30 min and included 2 presentations of the tone CS with an intertrial interval (ITI) of 8 min on average (range: 7 to 9 min). On day 2, rats received fear conditioning in context A which involved pairings of the tone CS with the foot shock US. Four, three or two pairings were used. The ITI was the same as that used for pre-exposure. On day 3, rats received fear extinction in context B during which the tone CS was presented 15 times in the absence of the foot shock US. The ITI ranged from 2 to 4 min and was 3 min on average. On day 4, rats received a post-extinction test that was identical to fear extinction. On days 5 and 6, rats received two retrieval tests. One tested the tone CS in the fear conditioning context A and the other in the fear extinction context B (order counterbalanced). Each test involved 10 presentations of the tone, except in one pharmacological experiment (Figure 5C; groups 2x) where the tone was only presented 5 times.

Optical silencing of BLA cholinergic terminals

During the pre-exposure sessions, rats were tethered to the patch cord, but no light was delivered. This was to ensure habituation to the procedure. To achieve optical silencing, optical fibers were connected to patch cords connected to an LED driver (Doric Lenses, Quebec, Canada). Orange light (625 nm; continuous; at least 8 mW at the tip of the fiber optics) was delivered for optical silencing of the BLA cholinergic terminals expressing eNpHR3.0. The light was activated at the start of the tone CS and ended 4 s after its termination.

Histology

At the end of the experiments, rats were rapidly anaesthetized with sodium pentobarbital (300 mg/kg; Vibrac Pty. Ltd., Sydney, Australia) and transcardially

perfused with cold 4% paraformaldehyde in 0.1 M sodium phosphate buffer (PB; pH 7.5). Brains were extracted and post-fixed in the same solution at 4°C overnight. Coronal 50 µm thick sections were cut through the targeted structures with a vibratome (VT1000, Leica Microsystems; Sydney, Australia) and stored at -30°C in a solution containing 30% ethylene glycol, 30% glycerol and 0.1 M sodium phosphate buffer, until they were processed for immunofluorescence.

To achieve immunodetection of cholinergic neurons, individual free-floating sections were rinsed three times for 10 min in Tris-buffered saline (TBS: 0.25 M Tris, 0.5 M NaCl, pH 7.5). After a 40 min incubation in 0.2% Triton X-100 in TBS, sections were rinsed three times in TBS. The slices were then incubated overnight at 4°C in TBS containing the goat anti-ChAT primary antibody (1/:300; Millipore; # AB144P). The following day, the slices were rinsed three times in TBS and incubated 60 min at room temperature with donkey anti-goat Cy3 (1:400; Jackson ImmunoResearch Laboratories; #705-165-147). After this incubation, sections were rinsed three times in TBS, mounted on Superfrost Plus-coated slides, and let dry for 10 min before being coverslipped in Vectashield mounting medium (Vector Laboratories, # VEH-1000). The rinsing and mounting procedure just described was used to examine spread of the viral infusions and accurate placement of the fiber optics.

Fluorescent brain sections were imaged using a spinning disk confocal system equipped with the Diskovery multi-modal imaging platform (SAR/Andor Technology) and a Zyla 4.2 sCMOS camera (Andor Technology), that allows fast capture of large mosaic images and high-sensitivity high-dynamic range confocal imaging. The Diskovery platform was mounted on a Nikon Eclipse TiE microscope body with a motorized stage and the Nikon Perfect Focus System, and image acquisition was controlled by Nikon NIS-Elements software. All images were acquired with a Nikon 20X objective (0.75 N.A. CFI Plan App). All images were then processed using Open Source ImageJ29Fiji software [2]. Viral expression specificity (Figures S1B, S1D and S1M) was achieved through manual cell counting and involved calculating a percentage of the total number of fluorophore-labeled (eYFP) cells relative to the total number of ChAT-positive cells, and a percentage of the total number of ChAT-positive cells relative to the total number of fluorophore-labeled (eYFP) cells. The spread of the viral infusions and the accurate placement of the fiber optics were performed by an experimenter unaware of the behavioral score underlying the samples. In all analyses, the experimenters used boundaries defined in the atlas of Paxinos and Watson [3]. 31 rats were excluded due to inadequate viral infusion spread or misplacement of the fiber optics.

Statistical analyses

All data presented met the assumptions of the statistical test used. Freezing was the index of conditioned fear. It was rated in a time-sampling manner and judged as either freezing or not freezing every 2 s by a trained observer blind to the subjects' group assignment. A proportion of the data was cross scored by a second naive observer; there was a high level of agreement between observers (Pearson product moment correlation > 0.9). Freezing was defined as the absence of all movements except those related to breathing and characterized by a rigid posture distinct from resting or sleeping. Freezing was scored during the baseline period (1 minute before the first tone CS presentation during fear conditioning, fear extinction and post-extinction test; 3 minutes before the first tone CS presentation during the retrieval tests) and the tone CS presentation period. For simplicity, freezing during test and the retrieval tests is

shown as the average of the first five tone trials in the main Figures. Data for all trials is shown in Supplemental Figures. The differences between groups were analyzed by means of planned orthogonal contrasts. For optogenetic experiments involving a four-group design (Figures 2, 4, S2 and S4), a first contrast tested for differences between the two eYFP groups (eYFP-ON and eYFP-OFF). A second contrast combined these two eYFP groups and tested for difference with the third control group eNpHR3.0-OFF. Finally, a third contrast combined the three control groups and tested for differences with the experimental eNpHR3.0-ON group. For optogenetic experiments involving a two-group design (Figures 3, S3, 5D and S5B), a single contrast tested for differences between the two groups. For pharmacological experiments involving a four-group design (Figures 5B and S5A), the contrasts used were akin a two-way ANOVA and used two main factors (training strength and treatment) and their interactions. Retrieval tests included the within-subject factor of context identity (i.e., context A vs. context B). Within-session changes in freezing were assessed by planned linear trend analyses. All these procedures and analyses have been described by Hays [4] and were conducted in the PSY software (School of Psychology, The University of New South Wales, Australia). The Type I error rate was controlled at alpha = 0.05 for each contrast tested. If interactions were detected, follow-up simple effects analyses were calculated to determine the source of the interactions.

Supplementary Figures

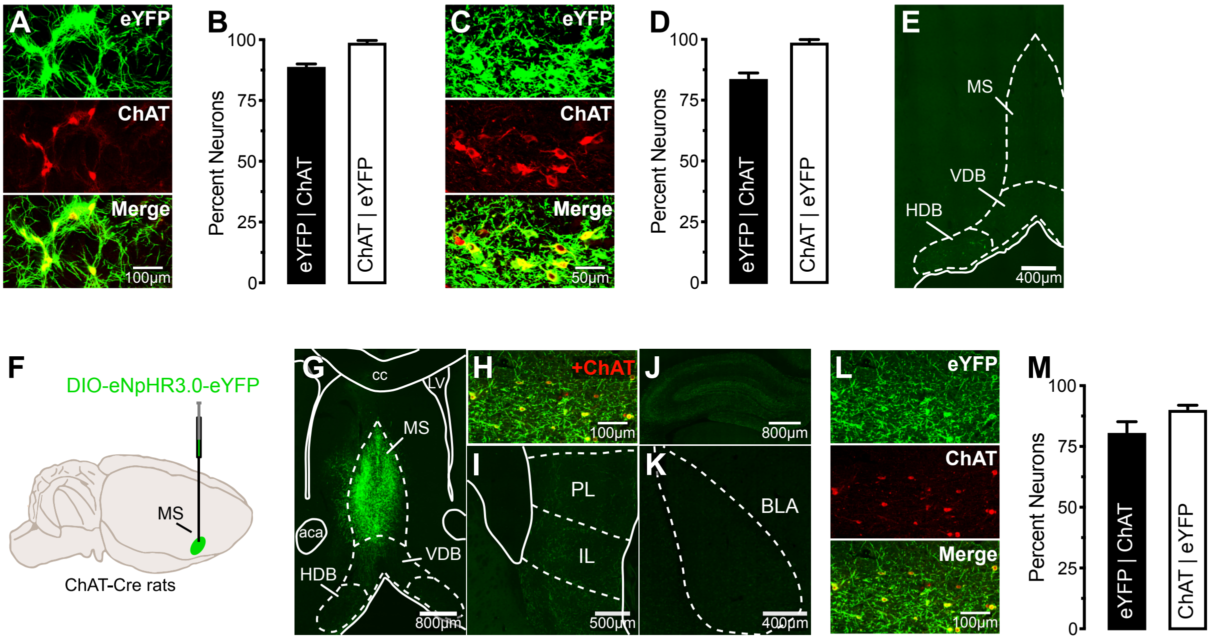

Figure S1 - Viral tracing in ChAT::Cre⁺ rats related to Figure 1
A. Individual channel and composite micrographs following NBM infusion of DIO-eNpHR3.0-eYFP. **B.** Most NBM cholinergic neurons were eYFP-positive (eYFP | ChAT; *n* = 4 rats; 85 ChAT neurons total) and most NBM eYFP-positive neurons were cholinergic (ChAT | eYFP; 77 eYFP-positive neurons total). **C**. Individual channel and composite micrographs following HDB infusion of DIO-eNpHR3.0-eYFP. **D**. Most HDB cholinergic neurons were eYFP-positive (eYFP | ChAT; *n* = 3 rats; 76 ChAT neurons total) and most HDB eYFP-positive neurons were cholinergic (ChAT | eYFP; 64 eYFP-positive neurons total). **E.** Micrograph showing eYFP-positive cholinergic neurons in the anterior basal forebrain following unilateral BLA infusion of Rg-DIO-eNpHR3.0-eYFP. eYFP-positive cholinergic neurons were concentrated in the HDB and none were detected in the VDB or MS. **F.** ChAT::Cre⁺ rats were unilaterally infused in the MS with DIO-eNpHR3.0-eYFP. **G.** Micrograph showing viral expression in MS neurons. **H.** MS viral expression was restricted to cholinergic neurons **I-K.** MS cholinergic neurons send projections to the PL, IL and dorsal hippocampus but not the BLA. **L.** Individual channel and composite micrographs following MS infusion of DIO-eNpHR3.0-eYFP. **M**. Most MS cholinergic neurons were eYFP-positive (eYFP | ChAT; *n* = 2 rats; 134 ChAT neurons total) and most NBM eYFP-positive neurons were cholinergic (ChAT | eYFP; 119 eYFP-positive neurons total).

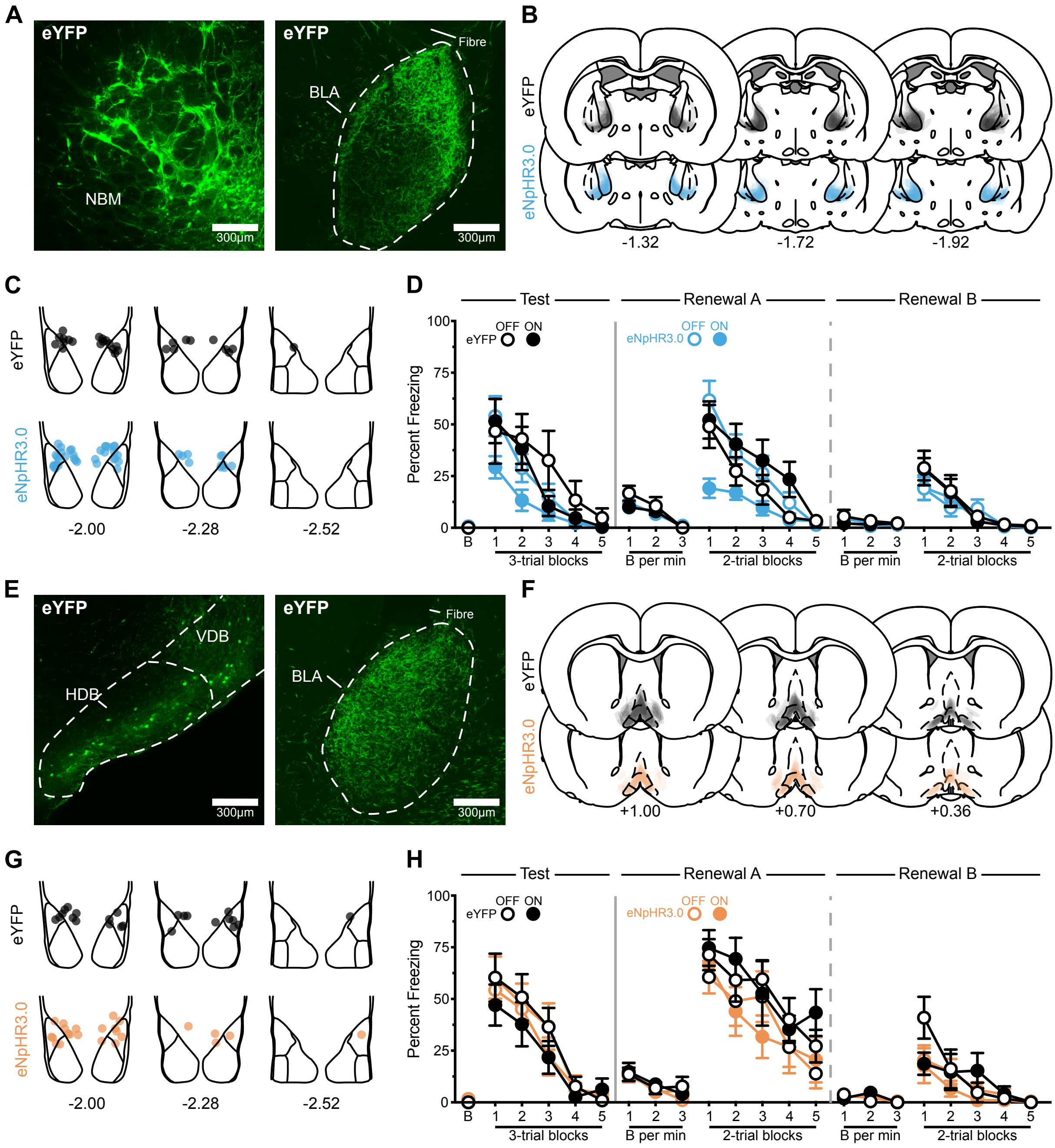

Figure S2 - Histological and behavioral data related to Figure 2

A. Micrographs showing eYFP viral expression in NBM cholinergic neurons (left), eYFP-positive BLA cholinergic terminals and fiber optic placements (right). **B.** Minimal (light black for eYFP and light blue for eNpHR3.0-eYFP) and maximal (darker black for eYFP and darker blue for eNpHR3.0-eYFP) extent of the NBM viral infection. Distances are indicated in mm from bregma. **C.** Location of fiber optics in the BLA (black for eYFP and blue for eNpHR3.0-eYFP). Distances are indicated in mm from bregma. **D.** ^{Ach}NBM"BLA silencing during fear conditioning reduced freezing at the post-extinction test ($F_{1,31}$=8.67, p<0.01) relative the control rats, which displayed equivalent freezing (smallest p=0.29). By the end of the test (last 3-trial block), all groups exhibited equivalent and low freezing (smaller p=0.12). During the retrieval tests, freezing was higher in context A than in context B ($F_{1,31}$=38.73, p<0.001). The control groups displayed equivalent freezing (smallest p=0.29), and this freezing was

reduced by ^{Ach}NBM"BLA silencing during fear conditioning ($F_{1,31}=8.67$, $p<0.01$). This silencing abolished renewal ($F_{1,31}=9.45$, $p<0.01$). Although the control groups froze more in context A than context B (eYFP-OFF: $F_{1,5}=20.19$, $p<0.01$; eYFP-ON: $F_{1,7}=16.41$, $p<0.01$; eNpHR3.0-OFF: $F_{1,9}=13.76$, $p<0.01$), rats that received ^{Ach}NBM"BLA silencing during fear conditioning froze very little in both contexts ($p=0.20$). All rats discriminated the two contexts, as baseline freezing (B) during the 3 minutes preceding the first CS presentation was higher in context A than in context B ($F_{1,31}=37.04$, $p<0.001$), irrespective of groups (smallest $p=0.55$). This difference was absent in the 1st minute preceding the first CS presentation ($p=0.06$), irrespective of groups (smallest $p=0.58$). **E.** Micrographs showing eYFP viral expression in HDB cholinergic neurons (left), eYFP-positive BLA cholinergic terminals and fiber optic placements (right). **F.** Minimal (light black for eYFP and light orange for eNpHR3.0-eYFP) and maximal (darker black for eYFP and darker orange for eNpHR3.0-eYFP) extent of the HDB viral infection. Distances are indicated in mm from bregma. **G.** Location of fiber optics in the BLA (black for eYFP and orange for eNpHR3.0-eYFP). Distances are indicated in mm from bregma. **H.** ^{Ach}HDB"BLA silencing during fear conditioning had no effect on freezing at the post-retrieval test ($p=0.43$) relative to the control groups, which displayed equivalent freezing (smallest $p=0.22$). By the end of the test (last 3-trial block), all groups exhibited equivalent and low freezing (smaller $p=0.10$). During the retrieval tests, freezing was higher in context A than in context B ($F_{1,31}=47.51$, $p<0.001$). All groups displayed similar freezing ($p=0.08$), and fear renewal was unaffected by ^{Ach}HDB"BLA silencing during fear conditioning ($p=0.08$). All rats froze more in context A than in context B (eYFP-OFF: $F_{1,6}=6.98$, $p<0.05$; eYFP-ON: $F_{1,4}=12.82$, $p<0.05$; eNpHR3.0-OFF: $F_{1,5}=14.11$, $p<0.05$; eNpHR3.0-ON: $F_{1,5}=23.79$, $p<0.01$). All rats discriminated between the two contexts, as baseline freezing (B) during the 3 minutes preceding the first CS presentation was higher in context A than in context B ($F_{1,20}=17.73$, $p<0.001$), irrespective of groups (smallest $p=0.50$). This difference was maintained in the 1st minute preceding the first CS presentation ($F_{1,20}=5.49$, $p<0.05$), irrespective of groups (smallest $p=0.53$).

Data are shown as mean ± SEM.

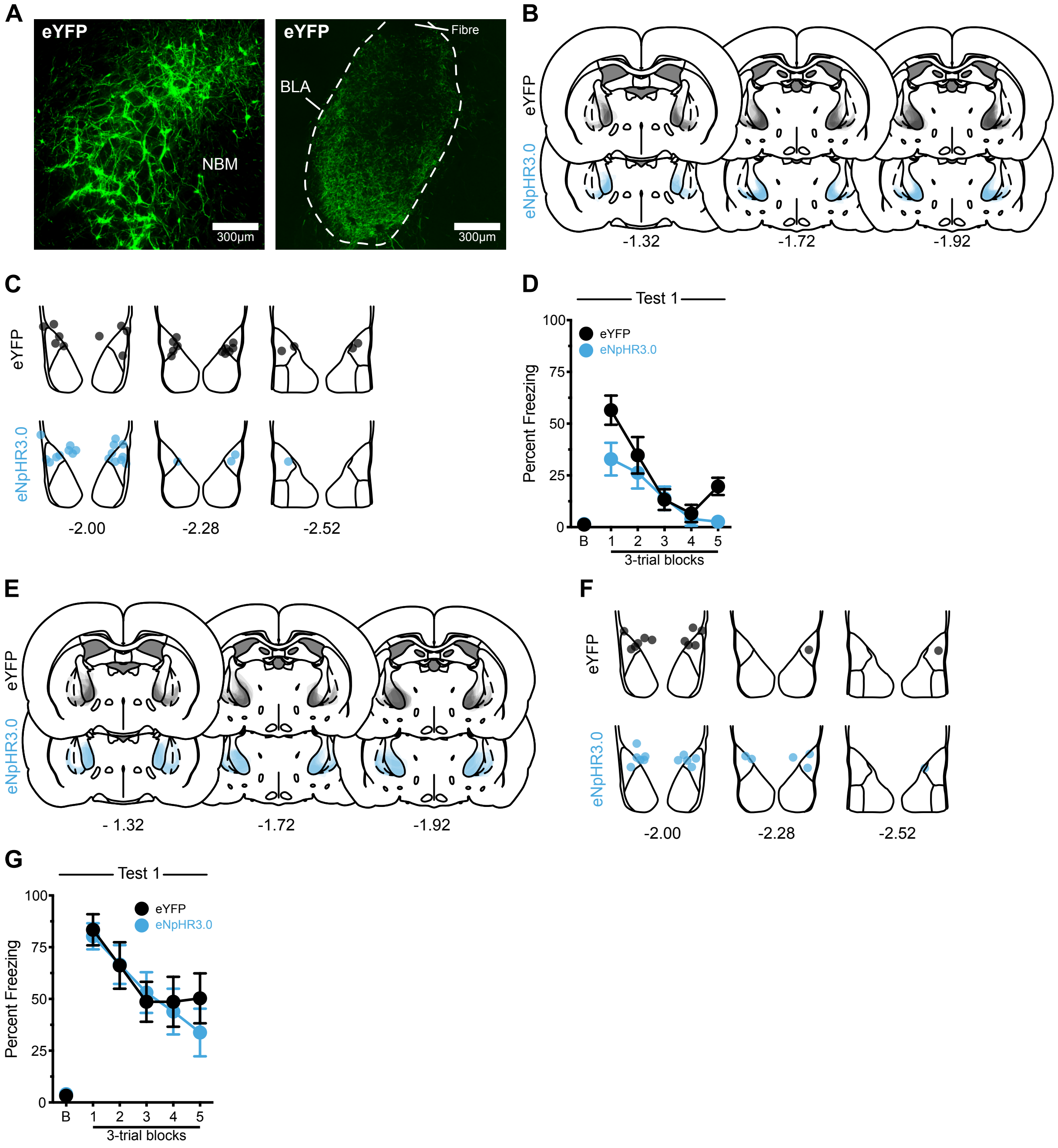

Figure S3 - Histological and behavioral data related to Figure 3
A. Micrographs showing eYFP viral expression in NBM cholinergic neurons (left), eYFP-positive BLA cholinergic terminals and fiber optic placements (right). **B.** Minimal (light black for eYFP and light blue for eNpHR3.0-eYFP) and maximal (darker black for eYFP and darker blue for eNpHR3.0-eYFP) extent of the NBM viral infection in rats submitted to the weak fear conditioning protocol. Distances are indicated in mm from bregma. **C.** Location of fiber optics in the BLA (black for eYFP and blue for eNpHR3.0-eYFP) of rats submitted to the weak fear conditioning protocol. Distances are indicated in mm from bregma. **D.** ^{Ach}NBM"BLA silencing during weak fear conditioning had no effect on freezing at test (p=0.23). **E.** Minimal (light black for eYFP and light blue for eNpHR3.0-eYFP) and maximal (darker black for eYFP and darker blue for eNpHR3.0-eYFP) extent of the NBM viral infection in rats submitted to the strong fear conditioning protocol. Distances are indicated in mm from bregma. **F.** Location of fiber optics in the

BLA (black for eYFP and blue for eNpHR3.0-eYFP) of rats submitted to the strong fear conditioning protocol. Distances are indicated in mm from bregma. **G.** ^{Ach}NBM"BLA silencing during strong fear conditioning had no effect on freezing at test 1 (p=0.72). Data are shown as mean ± SEM.

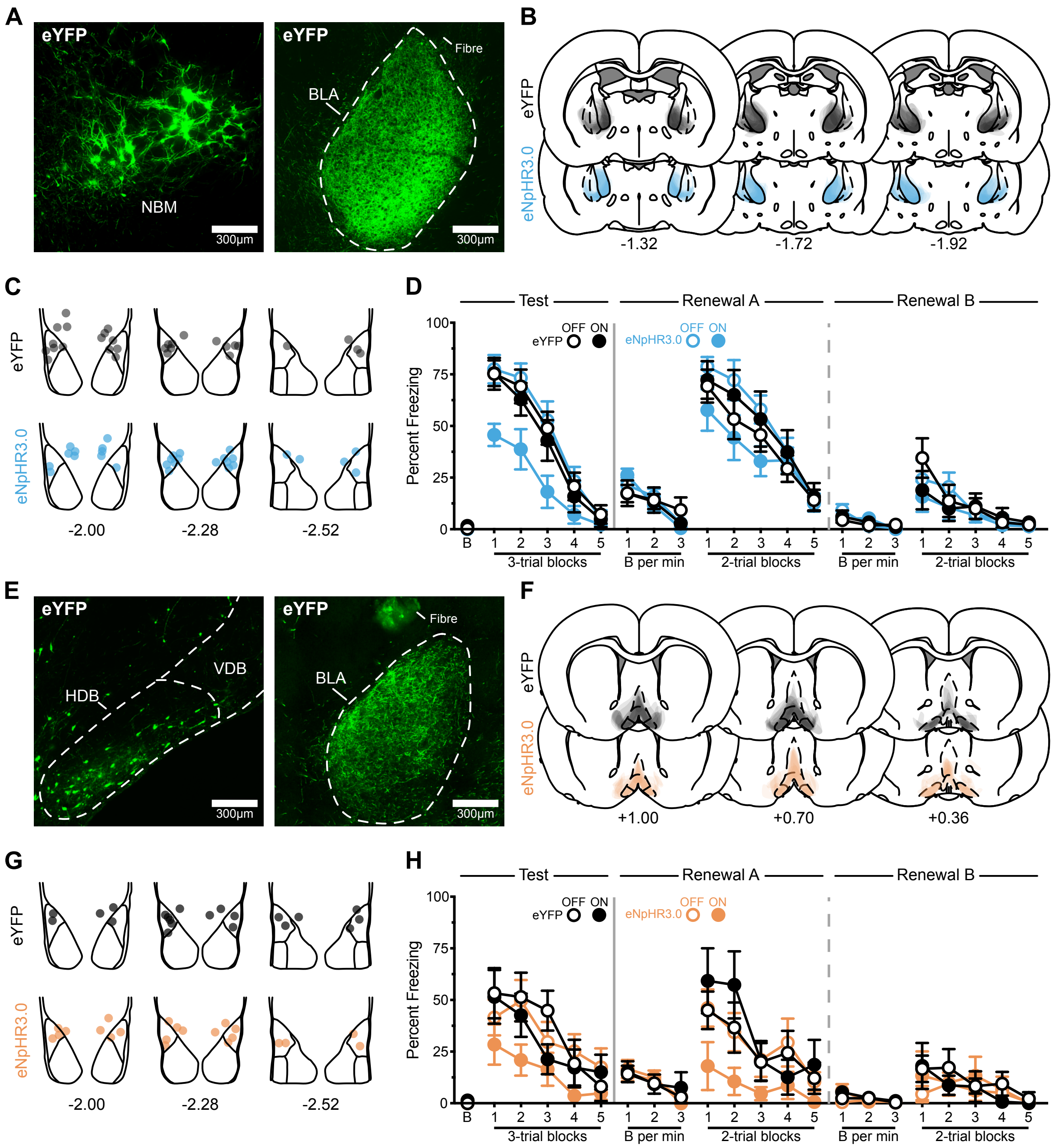

Figure S4 - Histological and behavioral data related to Figure 4
A. Micrographs showing eYFP viral expression in NBM cholinergic neurons (left), eYFP-positive BLA cholinergic terminals and fiber optic placements (right). **B.** Minimal (light black for eYFP and light blue for eNpHR3.0-eYFP) and maximal (darker black for eYFP and darker blue for eNpHR3.0-eYFP) extent of the NBM viral infection. Distances are indicated in mm from bregma. **C.** Location of fiber optics in the BLA (black for eYFP and blue for eNpHR3.0-eYFP). Distances are indicated in mm from bregma. **D.** ^{Ach}NBM"BLA silencing during fear extinction reduced freezing during the post-extinction test ($F_{1,26}$=28.52, p<0.001) relative to the control rats, which displayed equivalent freezing (smallest p=0.32). By the end of the test (last 3-trial block), all groups exhibited equivalent and low freezing (smaller p=0.38). During the retrieval tests, freezing was higher in context A than in context B ($F_{1,26}$=154.28, p<0.001). All

groups displayed similar freezing ($p=0.10$), and fear renewal was unaffected by ^{Ach}HDB"BLA silencing during fear conditioning ($p=0.30$). All rats froze more in context A than in context B (eYFP-OFF: $F_{1,8}=32.02$, $p<0.001$; eYFP-ON: $F_{1,5}=34.56$, $p<0.01$; eNpHR3.0-OFF: $F_{1,7}=79.99$, $p<0.001$; eNpHR3.0-ON: $F_{1,6}=24.48$, $p<0.01$). All rats discriminated the two contexts, as baseline freezing (B) during the 3 minutes preceding the first CS presentation was higher in context A than in context B ($F_{1,26}=15.73$, $p<0.001$), irrespective of groups (smallest $p=0.64$). This difference was absent in the 1st minute preceding the first CS presentation ($p=0.19$), irrespective of groups (smallest $p=0.39$). **E.** Micrographs showing eYFP viral expression in HDB cholinergic neurons (left), eYFP-positive BLA cholinergic terminals and fiber optic placements (right). **F.** Minimal (light black for eYFP and light orange for eNpHR3.0-eYFP) and maximal (darker black for eYFP and darker orange for eNpHR3.0-eYFP) extent of the HDB viral infection. Distances are indicated in mm from bregma. **G.** Location of fiber optics in the BLA (black for eYFP and orange for eNpHR3.0-eYFP). Distances are indicated in mm from bregma. **H.** ^{Ach}HDB"BLA silencing during fear extinction reduced freezing during the post-extinction test ($F_{1,18}=7.77$, $p<0.005$) relative to the control rats, which displayed equivalent freezing (smallest $p=0.45$). By the end of the test (last 3-trial block), all groups exhibited equivalent and low freezing (smaller $p=0.22$). During the retrieval tests, freezing was higher in the conditioning context A than in the extinction context B ($F_{1,18}=34.67$, $p<0.001$). The control groups displayed equivalent freezing (smallest $p=0.56$), and this freezing was left intact by ^{Ach}HDB"BLA silencing during fear extinction ($p=0.07$). This silencing abolished renewal ($F_{1,18}=11.07$, $p<0.01$). Although the control groups froze more in context A than context B (eYFP-OFF: $F_{1,5}=7.70$, $p<0.05$; eYFP-ON: $F_{1,4}=12.69$, $p<0.05$; eNpHR3.0: $F_{1,5}=19.89$, $p<0.01$), rats that received ^{Ach}NBM"BLA silencing during fear conditioning froze very little in both contexts ($p=0.95$). All rats discriminated the two contexts, as baseline freezing (B) during the 3 minutes preceding the first CS presentation was higher in context A than in context B ($F_{1,18}=29.48$, $p<0.001$), irrespective of groups (smallest $p=0.50$). This difference was absent in the 1st minute preceding the first CS presentation ($p=0.13$), irrespective of groups (smallest $p=0.25$).
Data are shown as mean ± SEM.

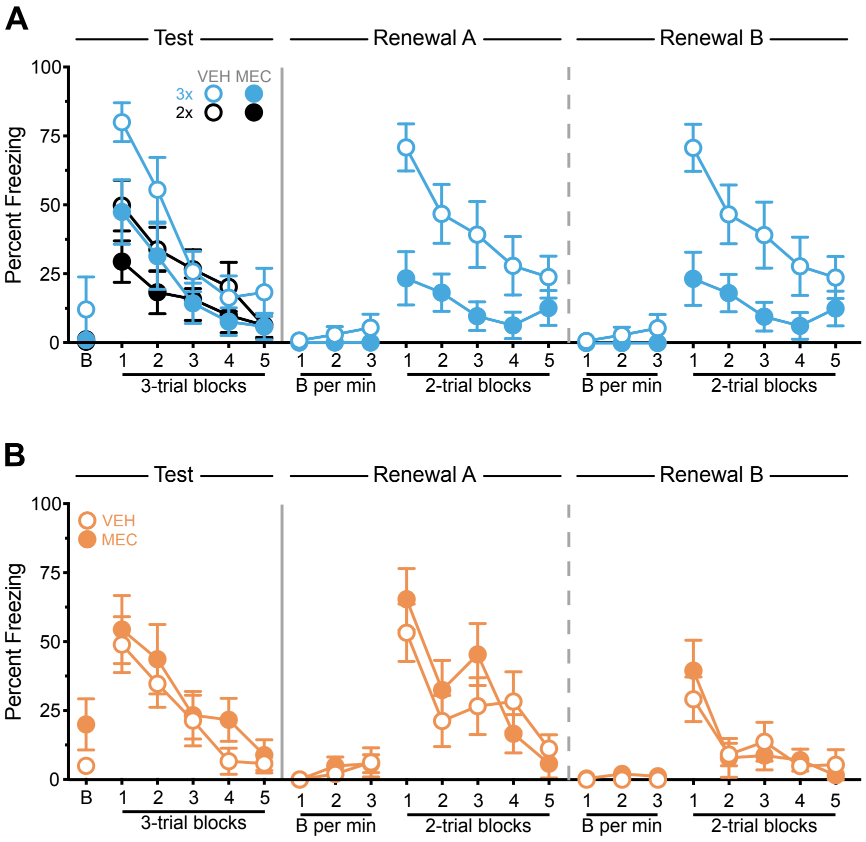

Figure S5 - Behavioral data related to Figure 5

A. Freezing during the post-extinction test was not influenced by the fear conditioning protocol (weak vs. strong), but it was reduced by MEC treatment given before conditioning ($F_{1,29}$=9.74, $p<0.01$), regardless of protocols ($p=0.49$). By the end of the test (last 3-trial block), all groups exhibited equivalent and low freezing (smaller $p=0.12$). During the retrieval tests in strongly conditioned rats, freezing was higher in context A than in context B ($F_{1,15}$=12.78, $p<0.01$). Freezing was reduced in MEC-treated rats ($F_{1,15}$=16.24, $p<0.001$) and renewal was influenced by the pharmacological treatment ($F_{1,15}$=4.61, $p<0.05$). Renewal was observed VEH-treated rats ($F_{1,7}$=10.11, $p<0.05$) but was absent in MEC-treated rats ($p=0.19$). Baseline freezing (B) during the 3 minutes preceding the first CS presentation was equivalent in context A and context B ($p=0.93$), irrespective of groups (smallest $p=0.20$). This lack of difference was preserved in the 1st minute preceding the first CS presentation ($p=0.92$), irrespective of groups (smallest $p=0.44$). Note that for simplicity, the baseline data for the weakly conditioned rats are not plotted. **B.** MEC before extinction had not effect on freezing at the post-extinction test ($p=0.37$). By the end of the test (last 3-trial block), all groups exhibited equivalent and low freezing (smaller $p=0.39$). During the retrieval tests, freezing was higher in context A than in context B ($F_{1,14}$=47.79, $p<0.001$), regardless of treatment ($p=0.83$). Baseline freezing (B) during the 3 minutes preceding the first CS presentation was equivalent in the context A and context B ($p=0.18$), irrespective of groups (smallest $p=0.91$). This lack of difference was preserved in the 1st minute preceding the first CS presentation ($p=0.13$), irrespective of groups (smallest $p=0.81$).

Data are shown as mean ± SEM.

Supplementary References

1 I.B. Witten, E.E. Steinberg, S.Y. Lee, T.J. Davidson, K.A. Zalocusky, M. Brodsky *et al.*, Recombinase-driver rat lines: tools, techniques, and optogenetic application to dopamine-mediated reinforcement., *Neuron* **72** (2011), pp. 721–733.

2 C.T. Rueden, J. Schindelin, M.C. Hiner, B.E. DeZonia, A.E. Walter, E.T. Arena *et al.*, ImageJ2: ImageJ for the next generation of scientific image data, *BMC bioinformatics* **18** (2017), pp. 529.

3 G. Paxinos and C. Watson, The rat brain in stereotaxic coordinates: hard cover edition, Elsevier, (2006).

4 W.L. Hays, Statistics for psychologists, Holt, Rinehart and Winston, New Yok (1963).
